# Comparative Effects of Donepezil and Tacrine on Recall-Related Exploratory Behavior in a Subacute Lipopolysaccharide-Induced Neuroinflammatory Model of Cognitive Impairment

**DOI:** 10.3390/biomedicines14061306

**Published:** 2026-06-09

**Authors:** Adrian-Florentin Dragomir, Aurelia Cristiana Barbu, Smaranda Stoleru, Aurelian Zugravu, Maria Carina Dumitrescu, George Bazar, Cristina Isabel Viorica Ghita, Silvia Fratea, Clara Maria Stoleru, Oana Andreia Coman, Ion Fulga

**Affiliations:** 1 Faculty of Medicine, “Carol Davila” University of Medicine and Pharmacy, 050474 Bucharest, Romania; adrian-florentin.dragomir@drd.umfcd.ro (A.-F.D.); aurelia-cristiana.barbu@drd.umfcd.ro (A.C.B.); maria-carina.dumitrescu@rez.umfcd.ro (M.C.D.); george.bazar@rez.umfcd.ro (G.B.); isabel.ghita@umfcd.ro (C.I.V.G.); oana.coman@umfcd.ro (O.A.C.); ion.fulga@umfcd.ro (I.F.); 2 Service Anesthesie-Reanimation, Groupe Hospitalier de la Pitié-Salpêtrière, CEDEX 13, 75651 Paris, France; silvia.fratea@aphp.fr; 3 Faculty of Medical Engineering, National University of Science and Technology Politehnica Bucharest, 060042 Bucharest, Romania; clara_maria.stoleru@stud.fim.upb.ro

**Keywords:** lipopolysaccharide, neuroinflammation, donepezil, tacrine, Y-maze, recall, exploratory behavior, Alzheimer’s disease, acetylcholinesterase inhibitors

## Abstract

**Background/Objectives:** Neuroinflammation is increasingly recognized as an important contributor to Alzheimer-like cognitive impairment. Lipopolysaccharide (LPS) is commonly used in experimental models to trigger systemic immune activation and behavioral alterations associated with neuroinflammation. This study aimed to validate a subacute LPS-induced model of recall-phase impairment and to compare the effects of donepezil and tacrine on recall-related exploratory behavior in rats. **Methods:** Male Wistar rats were tested in a two-trial Y-maze paradigm consisting of an acquisition trial followed by a recall trial 24 h later. In the validation experiment, rats received saline or LPS 1 mg/kg intraperitoneally for four consecutive days. In the intervention experiment, rats received saline, LPS, or LPS combined with donepezil 1 or 3 mg/kg or tacrine 3 or 5 mg/kg. The primary recall-phase outcome was the unknown/known arm time ratio (U/K time ratio). Additional outcomes included arm times, arm entries, U/K entry ratios, discrimination indices, and mean time per entry. **Results:** Repeated LPS administration significantly reduced the U/K time ratio, decreased time- and entry-based discrimination indices, reduced time spent in the unknown arm, and decreased unknown-arm entries, without significantly altering acquisition-phase behavior, total entries, or mean time per entry. In the intervention experiment, donepezil 1 mg/kg and tacrine 5 mg/kg significantly increased the U/K time ratio compared with LPS. Discrimination indices and entry-based measures further supported a treatment-related shift toward novelty-directed exploration, while total arm entries and mean time per entry were not significantly changed. **Conclusions:** Subacute LPS administration produced a measurable recall-phase exploratory impairment in the Y-maze. Donepezil and tacrine attenuated several components of this impairment, with partially distinct dose-related behavioral profiles.

## 1. Introduction

Alzheimer’s disease (AD) is a neurodegenerative form of dementia characterized by progressive cognitive decline, extracellular deposition of β-amyloid plaques, and intracellular accumulation of hyperphosphorylated tau protein in the form of neurofibrillary tangles [1]. Although the amyloid cascade hypothesis remains central to AD pathogenesis, growing evidence highlights the important role of neuroinflammation as both a triggering and an amplifying factor in disease progression [2,3]. In experimental research, lipopolysaccharide (LPS), an outer-membrane component of Gram-negative bacteria, is commonly used to trigger systemic immune activation and neuroinflammation-related responses in animal models [4].

Among the available LPS preparations, LPS derived from *Escherichia coli* O55:B5 represents a well-established experimental source of LPS with strong immunostimulatory activity [5]. Structurally, LPS is a glycolipid composed of a hydrophobic lipid A region and a carbohydrate chain, which includes the core oligosaccharide and the O-specific polysaccharide region [5,6]. Other *E. coli* LPS serotypes, such as O111:B4, O26:B6, O127:B8, and O128:B12, are also used experimentally; although they activate broadly similar TLR4-dependent pathways, structural differences may influence inflammatory potency and response kinetics [5,6,7].

In rat models, LPS administration provides a controlled framework for investigating neuroinflammation-associated cognitive impairment and Alzheimer-like pathological mechanisms [8]. While acute administration typically produces transient neuroinflammation and reversible cognitive deficits, repeated administration over several consecutive days may lead to a more sustained inflammatory response accompanied by progressive neuronal dysfunction and longer-lasting impairments in learning and memory [9,10]. With more prolonged exposure, chronic paradigms may further promote persistent microglial activation, oxidative stress, synaptic alterations, and broader neurodegenerative-like changes [8,11,12,13].

The dose of LPS used in rat models varies considerably according to the intended experimental outcome [4,14]. For systemic administration, intraperitoneal doses commonly range from low sub-milligram doses to several mg/kg, depending on whether the aim is to induce mild systemic inflammation, acute sickness-like responses, or more sustained neuroinflammatory changes. Lower doses, such as 0.1–0.5 mg/kg, are often used to elicit mild to moderate inflammatory responses, whereas doses around 1 mg/kg have been associated with more robust systemic inflammation and measurable neurobehavioral or cognitive alterations in experimental models [15,16,17]. Repeated systemic administration paradigms are frequently used to model sustained neuroinflammation and may better reflect persistent inflammatory conditions relevant to Alzheimer-like pathology [17,18].

LPS exerts its effects primarily through activation of the innate immune response via Toll-like receptor 4 (TLR4). Following TLR4 activation, downstream signaling pathways involving nuclear factor kappa B (NF-κB) and mitogen-activated protein kinases (MAPKs) promote the release of pro-inflammatory mediators [4,19]. Among these, interleukin-1β (IL-1β), interleukin-6 (IL-6), and tumor necrosis factor-alpha (TNF-α) play central roles in amplifying the neuroinflammatory response [4,20]. These cytokines can disrupt synaptic plasticity and neuronal communication and may further stimulate the expression of cyclooxygenase-2 (COX-2), a key enzyme involved in the synthesis of pro-inflammatory prostaglandins [4,20,21].

Acetylcholinesterase inhibitors such as donepezil and tacrine remain relevant pharmacological tools for investigating cognitive impairment associated with Alzheimer-like pathology and neuroinflammation. Both compounds enhance cholinergic neurotransmission by inhibiting cholinesterase activity, thereby increasing acetylcholine availability in brain regions involved in learning and memory [21,22]. In addition to their symptomatic pro-cholinergic effects, cholinesterase inhibitors may influence inflammatory responses through the cholinergic anti-inflammatory pathway, suggesting that their effects in LPS-induced models may extend beyond classical neurotransmitter modulation [10,22,23,24].

Donepezil is a selective, reversible acetylcholinesterase inhibitor with a favorable pharmacological and tolerability profile [25]. Experimental studies have reported that donepezil can attenuate LPS-induced inflammatory responses, while also improving cognitive performance in animal models of Alzheimer-like impairment [25,26,27,28]. These findings support the relevance of donepezil as a reference compound for evaluating cholinergic modulation under neuroinflammatory challenge conditions.

Tacrine, the first cholinesterase inhibitor approved for the treatment of AD, differs from donepezil by inhibiting both acetylcholinesterase and butyrylcholinesterase. Although its clinical use has been restricted by hepatotoxicity and tolerability concerns, tacrine remains pharmacologically relevant because of its broad cholinesterase-inhibitory profile and continued use as a reference scaffold in the development of multitarget anti-Alzheimer compounds [29,30,31].

Although LPS-induced inflammatory challenge is widely used to model inflammation-associated cognitive impairment [4,8,32], the behavioral consequences of subacute systemic LPS exposure during recall remain incompletely characterized. In particular, it remains unclear how inflammatory challenge modifies exploratory allocation between familiar and unfamiliar spatial contexts, or whether this pattern can be differentially modulated by donepezil and tacrine.

The present study therefore used repeated systemic LPS administration as an impairment-inducing model relevant to the neuroinflammatory component of Alzheimer-like cognitive dysfunction. We aimed to determine whether this subacute inflammatory challenge produces a recall-phase alteration detectable in a two-trial Y-maze paradigm with a 24 h interval and whether this behavioral pattern is modulated by cholinesterase inhibition. After behaviorally characterizing the LPS-induced phenotype, we compared donepezil and tacrine as pharmacologically distinct cholinesterase inhibitors. The analysis focused on recall-related exploratory organization, including time allocation, arm-entry patterns, U/K ratios, discrimination indices, and time-per-entry measures, in order to distinguish changes in arm-selection dynamics from changes in average visit duration.

## 2. Materials and Methods

Ethical approval for the study was obtained from the Ethics Committee of the “Carol Davila” University of Medicine and Pharmacy, Bucharest, Romania (authorization no. 13619 from 24 May 2024). Animal procedures complied with the applicable national legal requirements for scientific animal research and were authorized and supervised by the National Sanitary Veterinary and Food Safety Authority (authorization no. 9 from 5 July 2024).

### 2.1. Animals

Male Wistar rats, aged 6–8 weeks and weighing 200–300 g at the time of behavioral testing, were obtained from the animal breeding facility of the “Carol Davila” University of Medicine and Pharmacy, Bucharest, Romania. Before inclusion in the experiments, the animals underwent a minimum 7-day acclimatization period under local laboratory conditions. During the study, rats were kept in individual cages positioned next to one another in the same animal room. Food and water were provided ad libitum, and the housing environment included natural ventilation, 40–60% relative humidity, and the standard institutional light cycle. This arrangement avoided direct physical interaction between animals, while still allowing indirect sensory contact with conspecifics. The use of individual cages was intended to standardize pre-test housing conditions and reduce potential cage-mate-related effects on exploratory behavior during the experimental period.

The animal experiments were conducted and reported in line with the ARRIVE recommendations. All procedures were carried out in the Department of Pharmacology during the 10:00–17:00 interval. Each rat was assigned to a single experiment. Euthanasia was performed under general anesthesia 24 h after the completion of behavioral testing.

### 2.2. Experimental Design

The study was organized into two separate protocols.

(A) LPS validation protocol. Two experimental groups were used, with 9 rats assigned to each group:

1. C = control group: saline;
2. LPS 1 = lipopolysaccharide 1 mg/kg.

In the validation experiment, animals received one intraperitoneal injection per day for 4 consecutive days. Rats in the control group were administered saline, whereas rats in the LPS 1 group received lipopolysaccharide at a dose of 1 mg/kg. On days 1–3, treatment was administered according to the same daily schedule without behavioral testing. On day 4, the injection was given 2 h before the acquisition trial. The recall trial was carried out 24 h later, without additional drug administration.

(B) Pharmacological intervention protocol under LPS challenge. Six experimental groups were used, with 9 rats assigned to each group:

1. C = control group: saline + saline;
2. LPS 1 = lipopolysaccharide 1 mg/kg + saline;
3. D1 = lipopolysaccharide 1 mg/kg + donepezil 1 mg/kg;
4. D3 = lipopolysaccharide 1 mg/kg + donepezil 3 mg/kg;
5. T3 = lipopolysaccharide 1 mg/kg + tacrine 3 mg/kg;
6. T5 = lipopolysaccharide 1 mg/kg + tacrine 5 mg/kg.

**Figure 1:**
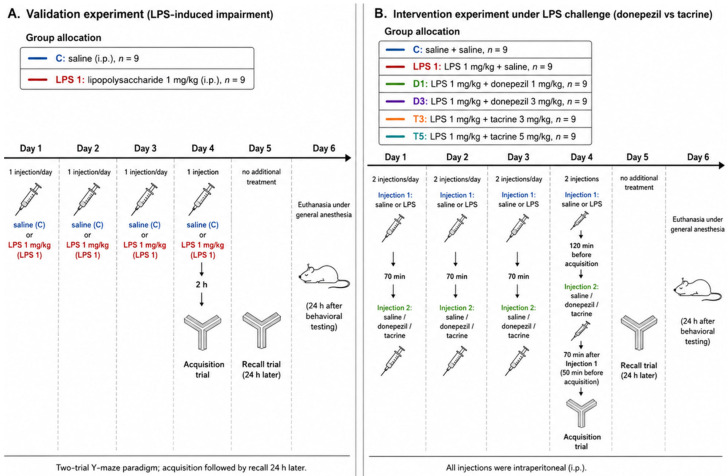
Experimental design and treatment timeline for the validation and intervention experiments. (**A**) Validation experiment: rats were allocated to C and LPS 1 groups, with 9 animals per group, and received one intraperitoneal injection per day for four consecutive days. The acquisition trial was performed 2 h after injection on day 4, followed by the recall trial 24 h later and euthanasia 24 h after behavioral testing. (**B**) Intervention experiment: rats were allocated to C, LPS 1, D1, D3, T3, and T5 groups, with 9 animals per group, and received two intraperitoneal injections per day for four consecutive days. On day 4, injection 1 was administered 120 min before acquisition, and injection 2 was administered 50 min before acquisition. The recall trial was performed 24 h later, followed by euthanasia 24 h after behavioral testing.

In the intervention experiment, all animals received two intraperitoneal injections per day for 4 consecutive days. The first daily injection consisted of lipopolysaccharide or saline, according to group allocation. Seventy minutes later, a second injection consisting of donepezil, tacrine, or saline was administered. On days 1–3, treatment was given according to the same schedule without behavioral testing. On day 4, the first injection was administered 120 min before the acquisition trial, whereas the second injection was administered 50 min before the acquisition trial. The recall trial was performed 24 h later, without additional treatment. The group allocation, treatment schedule, behavioral testing sequence, and euthanasia time point for both experimental protocols are summarized in Figure 1.

The number of animals per group was determined by considering previous Y-maze studies with comparable behavioral endpoints. Dose selection was guided by previous experimental studies and by our prior work on cholinergic modulation in Y-maze paradigms [33,34,35,36,37]. Donepezil doses of 1 and 3 mg/kg and tacrine doses of 3 and 5 mg/kg were selected to compare dose-related behavioral profiles of two pharmacologically distinct cholinesterase inhibitors under the same LPS-induced inflammatory challenge. These doses were not intended to establish a complete dose–response relationship, but to provide literature-supported treatment levels for each compound. The 4-day LPS regimen was chosen in accordance with earlier studies using repeated systemic administration to induce cognitive impairment in the context of neuroinflammatory challenge [38,39].

To minimize possible bias related to testing order, treatment administration and behavioral testing were scheduled according to a rotating group order. In the validation protocol, rats were alternated between the C and LPS 1 groups. In the intervention protocol, one rat from each group was treated and tested in the order C, LPS 1, D1, D3, T3, and T5 before the sequence was repeated for the next set of animals.

### 2.3. Drugs and Reagents

The compounds used in the present study were the following:

1. Lipopolysaccharide (LPS) from *Escherichia coli* O55:B5 (25 mg vials; Sigma-Aldrich/Merck, MO, USA; catalog no. L2880)
2. Donepezil hydrochloride (1 g vials; Sigma-Aldrich/Merck, MO, USA; catalog no. D6821; purity ≥ 98% by HPLC)
3. Tacrine (1 g vials; Sigma-Aldrich/Merck, MO, USA; catalog no. A79922; purity ≥ 99%)
4. 0.9% sodium chloride solution

All treatments were delivered by intraperitoneal injection, with the administered volume adjusted to 0.1 mL per 100 g of body weight.

### 2.4. Y-Maze Apparatus

A Y-maze task was used to evaluate spatial recognition memory. The apparatus consisted of three identical wooden arms arranged in a Y configuration; each arm had a length of 47 cm, a width of 15 cm, and a height of 38 cm. Adhesive foil was applied to the inner surfaces to provide a uniform texture throughout the maze.

The two-trial Y-maze paradigm was selected because it provides a rapid and relatively low-stress assessment of spatial recognition memory and novelty-directed exploratory behavior. The Y-maze is a simple and widely used behavioral paradigm in learning and memory studies, and two-trial versions allow assessment of exploration of a previously inaccessible novel arm [40]. Compared with more demanding behavioral paradigms such as the Morris water maze, the Y-maze requires minimal training and avoids exposure to swimming-related stress, which could potentially influence inflammatory and behavioral responses under repeated LPS administration [41]. Compared with novel object recognition, the present Y-maze protocol focuses on spatial-context novelty and exploratory allocation between familiar and unfamiliar arms, rather than object-directed exploration [42]. In addition, the two-trial Y-maze protocol allows evaluation of recall-phase exploratory allocation and discrimination-related behavioral indices within a relatively simple experimental design.

Lighting conditions were kept uniform throughout the maze, and environmental noise was reduced as much as possible during testing. The maze was placed in a constant position relative to external visual cues, which were kept unchanged across sessions in order to avoid spatial bias. Its orientation was checked regularly throughout the study.

A Logitech video camera (Lausanne, Switzerland) was placed beside the start arm to monitor Y-maze activity. Recordings were transmitted to a Lenovo computer (Morrisville, NC, USA) located outside the testing room, allowing the experimenter to observe the trial remotely. This setup provided continuous visualization of the maze and enabled reliable identification of arm entries. Video files were saved and subsequently evaluated offline to support blinded behavioral scoring and reduce observer-related bias.

### 2.5. Experimental Procedure

To minimize procedural variability, animal handling and placement in the maze were performed by the same experimenter throughout the study. After each rat was introduced into the maze, the experimenter exited the testing room. Rats were randomly distributed across groups, and video-based behavioral scoring was performed under blinded conditions.

The Y-maze procedure consisted of two 5 min trials: an acquisition trial and a recall trial performed 24 h later. During acquisition, only the start arm (S) and the familiar arm (K) were accessible, while the novel arm (U) remained closed with a wooden panel matching the maze walls and floor in texture and appearance. The familiar/novel arm assignment was alternated between left and right positions across rats. This distribution was kept balanced within each experimental group.

During the recall trial, rats had access to all arms, with the first-session familiar/novel configuration kept unchanged. Between trials, the maze was cleaned and allowed to dry to reduce the influence of residual odor cues.

### 2.6. Behavioral Analysis

Behavioral variables were analyzed from the recorded Y-maze sessions. For scoring, the maze was divided into the previously defined S, K, and U arms, together with the central intersection zone (I).

For the acquisition trial, the evaluated measures were S-arm time, K-arm time, S-arm entries, K-arm entries, and total entries. For the 24 h recall trial, the analysis focused on K- and U-arm exploration time and entry counts.

Derived recall-phase parameters were calculated separately for each animal. The U/K time ratio was defined as U-arm time divided by K-arm time, and the U/K entry ratio was computed as U-arm entries divided by K-arm entries. Mean time per entry (MTPE) was calculated for each arm, and time- and entry-based discrimination indices were calculated as (U − K)/(U + K).

Arm transitions were scored using the hind paws as reference points: entry required both hind paws to be inside an arm, whereas exit was recorded after both hind paws had left the arm.

Higher U/K ratios were interpreted as indicating a stronger recall-phase preference for the U arm. MTPE was included as an additional descriptor of visit duration.

### 2.7. Statistical Analysis

Statistical analysis was carried out using IBM SPSS Statistics for Windows, version 29.0 (IBM Corp., Armonk, NY, USA). Data are reported as mean ± standard deviation (SD). Variables from the acquisition and recall sessions were evaluated independently.

The primary endpoint for the recall phase was the U/K time ratio. The other recall-related measures, including the U/K entry ratio, time- and entry-based discrimination indices, absolute arm times, arm-entry counts, and MTPE, were used as secondary supportive outcomes.

Group comparisons were made using one-way ANOVA in both experimental protocols because the study design involved comparisons among independent experimental groups. In the validation experiment, one-way ANOVA was used to compare the C and LPS 1 groups. In the intervention experiment, whenever ANOVA indicated a significant group effect, Dunnett’s post hoc test was used for comparisons against the LPS 1 group, because the main objective was to determine whether donepezil or tacrine modified the behavioral effects induced by LPS.

Normality was checked within each group using the Shapiro–Wilk test, and equality of variances was examined with Levene’s test in order to verify the assumptions required for parametric group comparisons. Kruskal–Wallis testing was additionally used as a non-parametric sensitivity analysis and confirmed the same general pattern of results.

Potential outliers were assessed for all behavioral variables included in the statistical analysis using boxplot inspection, the 1.5 × interquartile range (IQR) rule, and standardized values calculated within each experimental group. Values flagged by IQR-based inspection were further evaluated using standardized values and biological plausibility. No observation exceeded the conventional standardized-value threshold for outliers (|z| > 3), and no data points were excluded from any analysis.

A power analysis was performed for the primary outcome measure (U/K time ratio). Based on the observed ANOVA effect size in the intervention experiment (η^2^ = 0.334; Cohen’s f = 0.708), with α = 0.05, statistical power set at 0.80, and six experimental groups, the estimated minimum sample size was approximately six animals per group. Therefore, the sample size used in the present study (n = 9 per group) was considered sufficient to detect the observed primary effect.

A *p* value < 0.05 was considered statistically significant.

## 3. Results

### 3.1. Model Validation

#### 3.1.1. Acquisition-Phase Behaviour (Session 1)

Table 1 summarizes acquisition-trial behavior.

No significant differences were detected between the two groups during Session 1 for S-arm time (F(1,16) = 0.42, *p* = 0.5283), K-arm time (F(1,16) = 0.75, *p* = 0.3985), S-arm entries (F(1,16) = 0.014, *p* = 0.9073), K-arm entries (F(1,16) = 3.16, *p* = 0.0945), or total entries (F(1,16) = 1.75, *p* = 0.2044). Overall, acquisition-phase exploratory behavior was similar between the two groups.

#### 3.1.2. U/K Time Ratio

**Figure 2:**
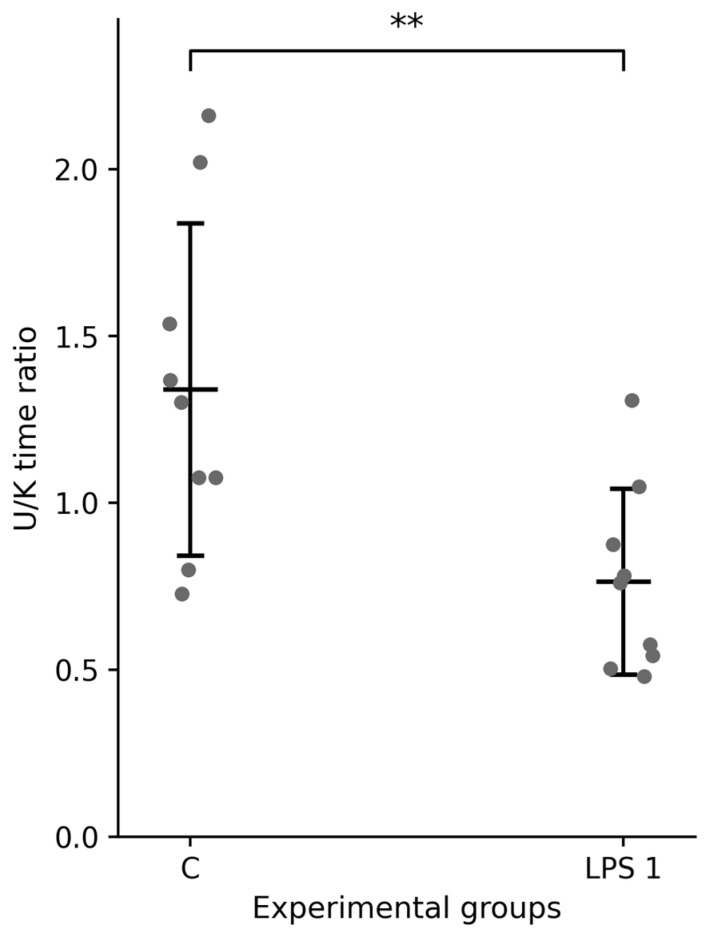
Recall-phase U/K time ratio shown as individual data points with mean ± SD (n = 9 per group). C = control group; LPS 1 = lipopolysaccharide 1 mg/kg. The bracket indicates the group comparison, and asterisks denote statistical significance (** *p* < 0.01).

For the validation experiment, recall performance was evaluated primarily through the U/K time ratio, which reflects how exploration was distributed between the novel and familiar arms. Additional measures, including absolute arm times, arm-entry counts, the U/K entry ratio, discrimination indices, and mean time per entry, were analyzed to support interpretation of the recall-phase behavioral profile. Figure 2 presents the recall-session values for the U/K time ratio.

The U/K time ratio differed significantly between groups (one-way ANOVA: F(1,16) = 9.20, *p* = 0.0079). These findings indicate that repeated LPS administration reduced allocation of recall-phase exploration toward the novel arm compared with the control group.

#### 3.1.3. Discrimination Indices

**Figure 3:**
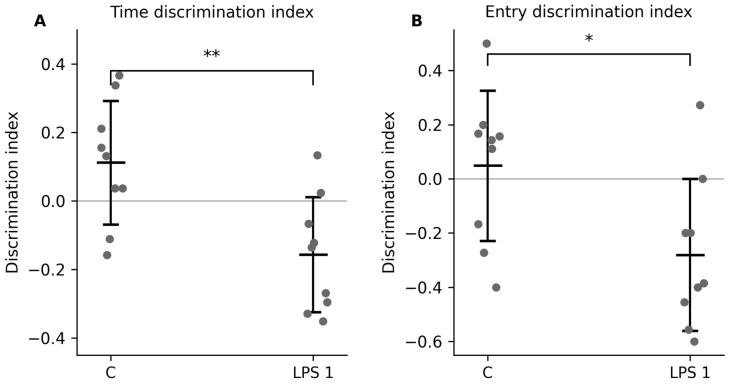
Time- and entry-based discrimination indices during the recall trial shown as individual data points with mean ± SD (n = 9 per group). (**A**) Time discrimination index. (**B**) Entry discrimination index. C = control group; LPS 1 = lipopolysaccharide 1 mg/kg. Group comparisons are shown with brackets, and significance levels are indicated by asterisks (* *p* < 0.05, ** *p* < 0.01).

The time-based discrimination index was significantly affected by treatment (one-way ANOVA: F(1,16) = 10.69, *p* = 0.0048). Mean values were 0.112 ± 0.181 for controls and −0.157 ± 0.168 for the LPS 1 group. A significant difference was also observed for the entry-based discrimination index (F(1,16) = 6.25, *p* = 0.0237), with mean values of 0.049 ± 0.278 in the control group and −0.280 ± 0.281 in the LPS 1 group. These results further indicate that repeated LPS exposure was associated with a reduced preference for the novel arm during recall. The time- and entry-based discrimination indices from the validation experiment are presented graphically in Figure 3A,B.

#### 3.1.4. Time Allocation Between the K and U Arms During Recall

**Figure 4:**
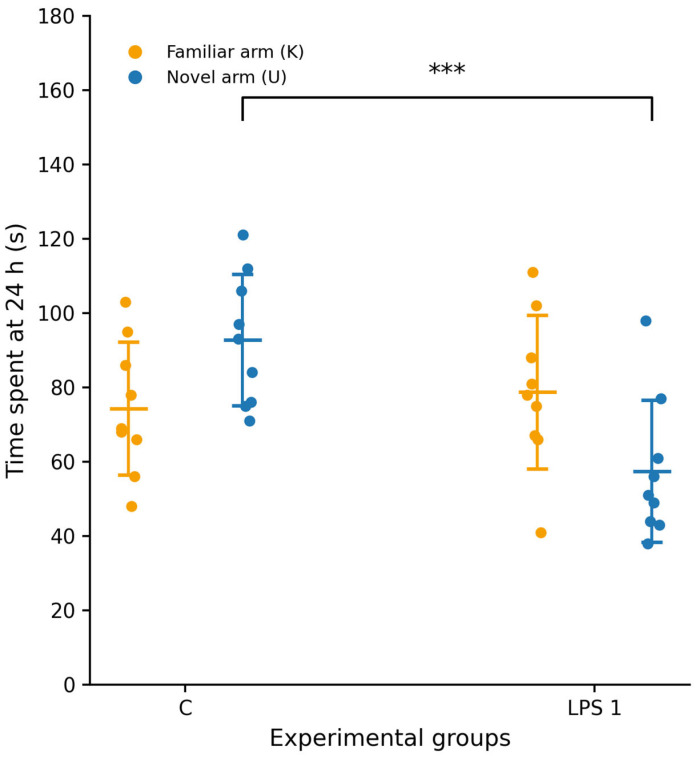
Time spent in the U and K arms during the recall trial, shown as individual data points with mean ± SD (n = 9 per group). C = control group; LPS 1 = lipopolysaccharide 1 mg/kg; K = familiar arm; U = novel arm. The bracket indicates the comparison between U-arm values, and asterisks denote statistical significance (*** *p* < 0.001).

Figure 4 presents the time allocation between the K and U arms during recall. No significant between-group difference was observed for time in the K arm (one-way ANOVA: F(1,16) = 0.24, *p* = 0.6323). By contrast, time spent in the U arm was significantly affected by treatment (F(1,16) = 16.56, *p* = 0.0009), with the LPS 1 group showing reduced exploration of the novel arm compared with controls. These findings are consistent with a reduction in novelty-directed exploration after repeated LPS exposure.

#### 3.1.5. Recall-Phase Entry Allocation Between the K and U Arms

**Figure 5:**
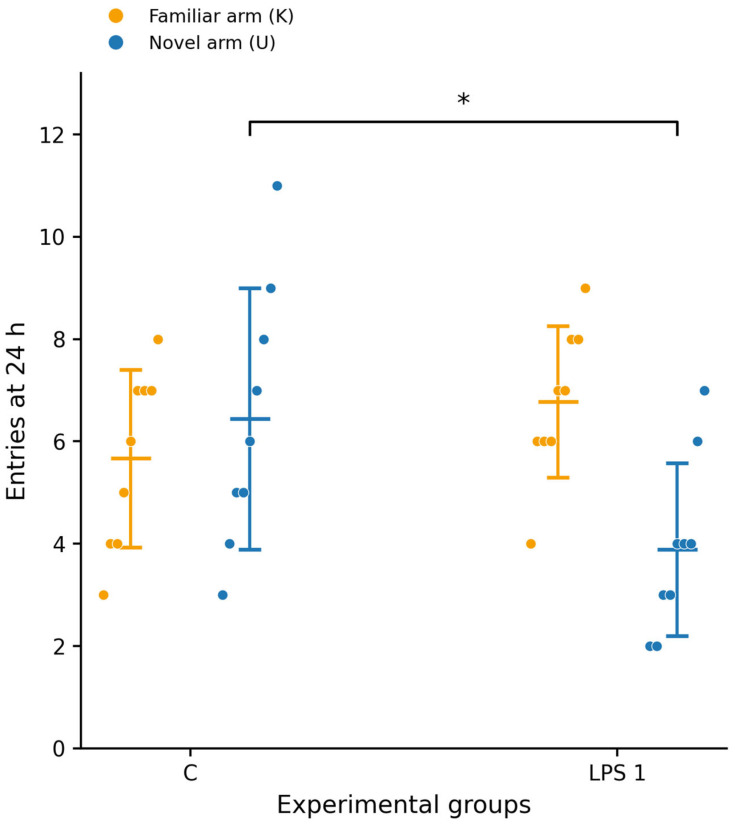
K- and U-arm entries during the recall trial, shown as individual data points with mean ± SD (n = 9 per group). C = control group; LPS 1 = lipopolysaccharide 1 mg/kg; K = familiar arm; U = novel arm. The bracket indicates the comparison between U-arm values, and the asterisk denotes statistical significance (* *p* < 0.05).

Recall-session entries into the K and U arms are shown in Figure 5. Entries into the K arm did not differ significantly between groups (one-way ANOVA: F(1,16) = 2.14, *p* = 0.1630). By contrast, entries into the U arm were significantly affected by treatment (F(1,16) = 6.26, *p* = 0.0236), with the LPS 1 group showing fewer entries into the U arm than the control group. Total arm entries were similar between groups (F(1,16) = 1.81, *p* = 0.1970). These findings indicate that repeated LPS administration reduced exploratory allocation toward the novel arm during recall, without a significant change in overall arm-entry activity.

#### 3.1.6. U/K Entry Ratio

**Figure 6:**
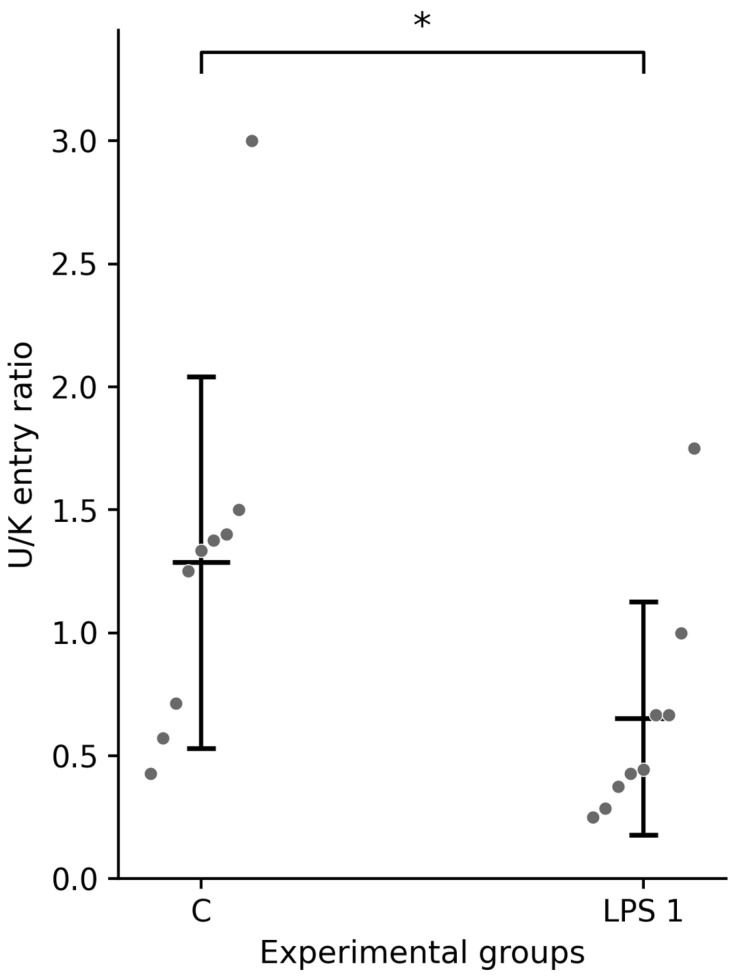
Recall-phase U/K entry ratio shown as individual data points with mean ± SD (n = 9 per group). C = control group; LPS 1 = lipopolysaccharide 1 mg/kg. The bracket indicates the group comparison, and the asterisk denotes statistical significance (* *p* < 0.05).

U/K entry ratio values during recall are shown in Figure 6. This ratio differed significantly between groups (one-way ANOVA: F(1,16) = 4.54, *p* = 0.0490), with lower values in the LPS 1 group than in controls. This result supports reduced entry-based exploration of the novel arm after repeated LPS exposure.

#### 3.1.7. Mean Time per Entry

Table 2 shows the MTPE for the K and U arms during recall. No significant between-group difference was found for mean time per entry in the K arm (one-way ANOVA: F(1,16) = 0.54, *p* = 0.4722) or in the U arm (F(1,16) = 0.04, *p* = 0.8401). These findings suggest that repeated LPS administration did not significantly affect average dwell time per visit in either arm during recall.

### 3.2. Donepezil and Tacrine Effects Under LPS Challenge

#### 3.2.1. Acquisition-Phase Behavior (Session 1)

Acquisition-phase behavioral parameters are presented in Table 3. Acquisition-phase variables were comparable across groups, including S-arm time (one-way ANOVA: F(5,48) = 0.16, *p* = 0.9752), K-arm time (F(5,48) = 0.34, *p* = 0.8880), S-arm entries (F(5,48) = 0.51, *p* = 0.7706), K-arm entries (F(5,48) = 0.53, *p* = 0.7556), total exploration time (F(5,48) = 0.36, *p* = 0.8718), and total arm entries (F(5,48) = 0.32, *p* = 0.8964). These findings indicate comparable acquisition-phase exploratory behavior across all experimental groups before recall testing.

#### 3.2.2. U/K Time Ratio

**Figure 7:**
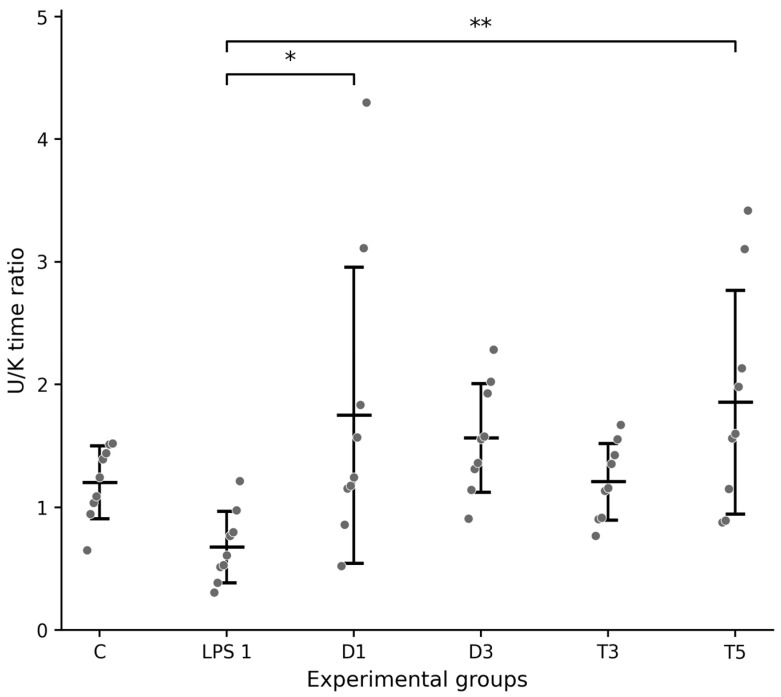
U/K time ratio during the recall trial under LPS challenge, shown as individual data points with mean ± SD (n = 9 per group). C = control group; LPS 1 = lipopolysaccharide 1 mg/kg + saline; D1 = lipopolysaccharide 1 mg/kg + donepezil 1 mg/kg; D3 = lipopolysaccharide 1 mg/kg + donepezil 3 mg/kg; T3 = lipopolysaccharide 1 mg/kg + tacrine 3 mg/kg; T5 = lipopolysaccharide 1 mg/kg + tacrine 5 mg/kg. Brackets show the relevant comparisons, and asterisks indicate statistical significance (* *p* < 0.05, ** *p* < 0.01).

In the intervention experiment, the main recall-related measure was the U/K time ratio, used to compare exploration of the U arm relative to the K arm. Absolute arm times, arm-entry counts, U/K entry ratio, discrimination indices, and mean time per entry were evaluated as complementary outcomes to describe the treatment-related behavioral pattern. Figure 7 shows the U/K time ratio values recorded during recall.

The U/K time ratio showed a significant overall difference among groups (one-way ANOVA: F(5,48) = 3.75, *p* = 0.0061). Dunnett’s post hoc analysis, performed against the LPS 1 group, showed higher ratio values in the D1 group (*p* = 0.0178) and T5 group (*p* = 0.0070). The comparisons between LPS 1 and C (*p* = 0.5716), D3 (*p* = 0.0780), or T3 (*p* = 0.5618) did not reach statistical significance.

These results indicate that, under LPS challenge, donepezil 1 mg/kg and tacrine 5 mg/kg increased novelty-directed exploration during recall, as reflected by higher U/K time ratio values.

#### 3.2.3. Discrimination Indices

Analysis of the time discrimination index showed a significant group effect (one-way ANOVA: F(5,48) = 6.52, *p* = 0.000106). The corresponding mean values were 0.076 ± 0.137 for C, −0.222 ± 0.202 for LPS 1, 0.169 ± 0.287 for D1, 0.199 ± 0.140 for D3, 0.078 ± 0.132 for T3, and 0.239 ± 0.224 for T5. In Dunnett’s comparisons against LPS 1, significantly higher values were found for C, D1, D3, T3, and T5. The corresponding *p* values, in the same order, were 0.0095, 0.0005, 0.0002, 0.0090, and <0.0001, respectively.

A significant overall effect was also found for the entry discrimination index (F(5,48) = 8.42, *p* = 8.95 × 10^−6^). Mean values were −0.016 ± 0.157 for C, −0.276 ± 0.197 for LPS 1, 0.194 ± 0.187 for D1, 0.172 ± 0.258 for D3, 0.152 ± 0.227 for T3, and 0.287 ± 0.217 for T5. Relative to LPS 1, significantly higher values were observed for C, D1, D3, T3, and T5, with *p* values of 0.0460, 0.000098, 0.0002, 0.0004, and <0.0001, respectively.

Overall, the treatment groups showed a more novelty-oriented recall profile than the LPS 1 group, consistent with attenuation of the LPS-related exploratory deficit.

**Figure 8:**
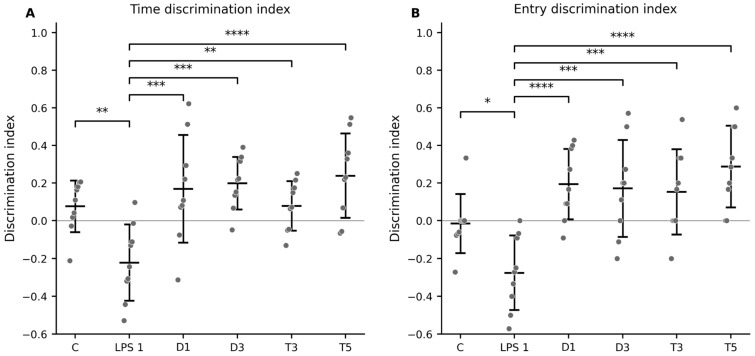
Time- and entry-based discrimination indices during the recall trial, shown as individual data points with mean ± SD (n = 9 per group). (**A**) Time discrimination index. (**B**) Entry discrimination index. C = control group; LPS 1 = lipopolysaccharide 1 mg/kg + saline; D1 = lipopolysaccharide 1 mg/kg + donepezil 1 mg/kg; D3 = lipopolysaccharide 1 mg/kg + donepezil 3 mg/kg; T3 = lipopolysaccharide 1 mg/kg + tacrine 3 mg/kg; T5 = lipopolysaccharide 1 mg/kg + tacrine 5 mg/kg. Brackets indicate the relevant group comparisons, and asterisks denote statistical significance (* *p* < 0.05, ** *p* < 0.01, *** *p* < 0.001, **** *p* < 0.0001).

The time- and entry-based discrimination indices from the intervention experiment are presented graphically in Figure 8A,B.

#### 3.2.4. Time Allocation Between the K and U Arms During Recall

**Figure 9:**
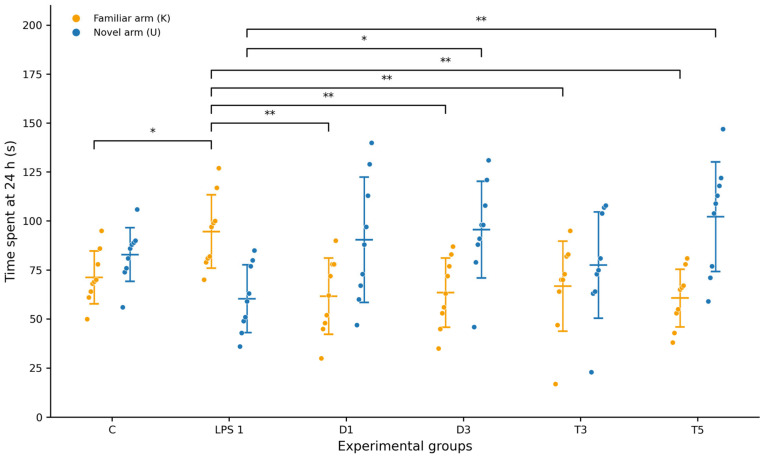
Time spent in the K and U arms during the recall trial, shown as individual data points with mean ± SD (n = 9 per group). C = control group; LPS 1 = lipopolysaccharide 1 mg/kg + saline; D1 = lipopolysaccharide 1 mg/kg + donepezil 1 mg/kg; D3 = lipopolysaccharide 1 mg/kg + donepezil 3 mg/kg; T3 = lipopolysaccharide 1 mg/kg + tacrine 3 mg/kg; T5 = lipopolysaccharide 1 mg/kg + tacrine 5 mg/kg. K = familiar arm; U = novel arm. Brackets indicate comparisons performed within the same arm across experimental groups, and asterisks denote statistical significance (* *p* < 0.05, ** *p* < 0.01).

Figure 9 shows recall-session time allocation between the K and U arms.

For K-arm time, one-way ANOVA showed a significant overall group effect (F(5,48) = 4.51, *p* = 0.0019). Mean values were 71.22 ± 13.48 s in the control group, 94.67 ± 18.70 s in the LPS 1 group, 61.67 ± 19.38 s in the D1 group, 63.44 ± 17.71 s in the D3 group, 66.78 ± 22.99 s in the T3 group, and 60.67 ± 14.65 s in the T5 group. Dunnett’s test was then applied with LPS 1 as the comparator. K-arm time was significantly lower in C, D1, D3, T3, and T5, with corresponding *p* values of 0.0348, 0.0015, 0.0028, 0.0088, and 0.0011, respectively.

For U-arm time, one-way ANOVA also showed a significant group effect (F(5,48) = 3.28, *p* = 0.0126). Mean values were 82.89 ± 13.74 s in the control group, 60.33 ± 17.28 s in the LPS 1 group, 90.44 ± 31.95 s in the D1 group, 95.56 ± 24.69 s in the D3 group, 77.56 ± 27.17 s in the T3 group, and 102.22 ± 28.04 s in the T5 group. Relative to LPS 1, Dunnett’s test showed significantly higher values in D3 (*p* = 0.0168) and T5 (*p* = 0.0034), whereas the comparisons with C, D1, and T3 were not statistically significant.

Overall, these findings indicate that the LPS-related redistribution of recall-phase exploration was attenuated by treatment, with the clearest shift toward the novel arm observed in the D3 and T5 groups.

#### 3.2.5. Recall-Phase Entry Allocation Between the K and U Arms

**Figure 10:**
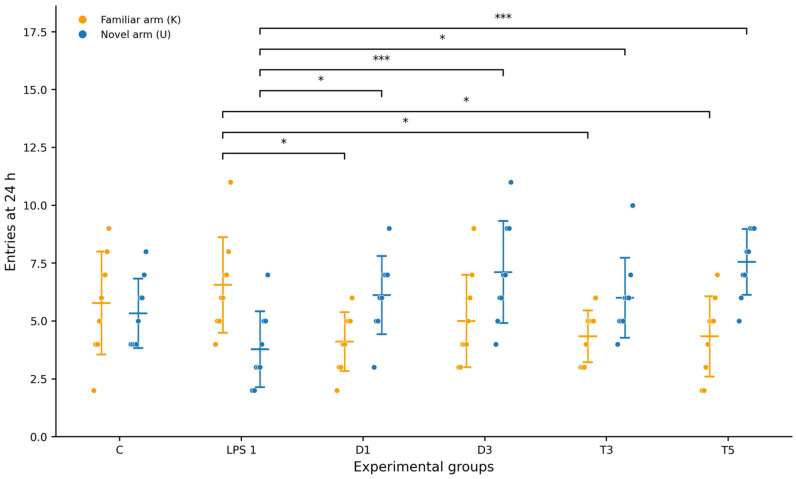
K- and U-arm entries during the recall trial, shown as individual data points with mean ± SD (n = 9 per group). C = control group; LPS 1 = lipopolysaccharide 1 mg/kg + saline; D1 = lipopolysaccharide 1 mg/kg + donepezil 1 mg/kg; D3 = lipopolysaccharide 1 mg/kg + donepezil 3 mg/kg; T3 = lipopolysaccharide 1 mg/kg + tacrine 3 mg/kg; T5 = lipopolysaccharide 1 mg/kg + tacrine 5 mg/kg. K = familiar arm; U = novel arm. Brackets indicate comparisons performed within the same arm across experimental groups, and asterisks denote statistical significance (* *p* < 0.05, *** *p* < 0.001).

Figure 10 shows recall-session entry counts for the K and U arms.

For K-arm entries, the ANOVA result was significant (F(5,48) = 2.66, *p* = 0.0335). Mean values were 5.78 ± 2.22 for C, 6.56 ± 2.07 for LPS 1, 4.11 ± 1.27 for D1, 5.00 ± 2.00 for D3, 4.33 ± 1.12 for T3, and 4.33 ± 1.73 for T5. Post hoc testing against LPS 1 showed lower K-arm entry counts in D1, T3, and T5, with corresponding *p* values of 0.0233, 0.0451, and 0.0451, respectively. The comparisons with C and D3 did not reach statistical significance.

U-arm entries also varied significantly across groups (one-way ANOVA: F(5,48) = 5.52, *p* = 0.0004). Mean values were 5.33 ± 1.50 for C, 3.78 ± 1.64 for LPS 1, 6.11 ± 1.69 for D1, 7.11 ± 2.20 for D3, 6.00 ± 1.73 for T3, and 7.56 ± 1.42 for T5. Compared with LPS 1, U-arm entry counts were higher in D1, D3, T3, and T5, with *p* values of 0.0249, 0.0007, 0.0352, and 0.0002, respectively. The C versus LPS 1 comparison was not significant (*p* = 0.2129).

Total arm entries were comparable across groups (F(5,48) = 0.98, *p* = 0.4414).

Overall, these results indicate that the reduction in novelty-directed arm selection induced by LPS was attenuated by treatment, without a significant overall change in total entry counts.

#### 3.2.6. U/K Entry Ratio

**Figure 11:**
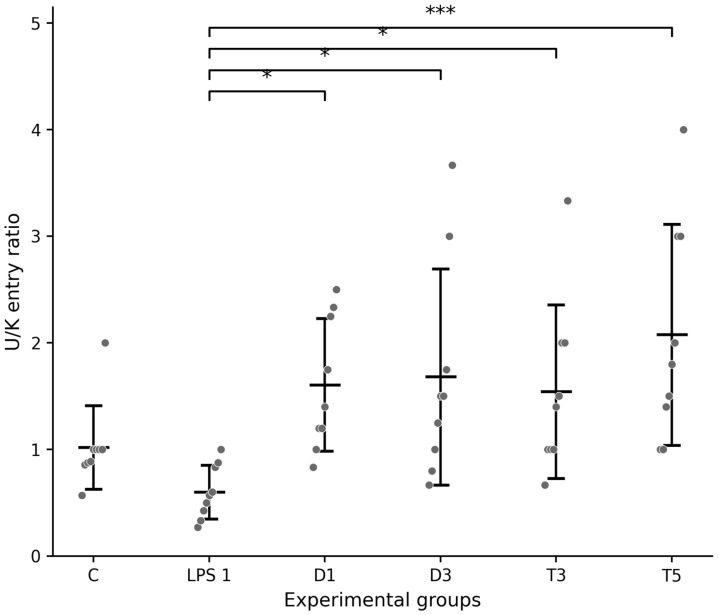
U/K entry ratio during the recall trial, shown as individual data points with mean ± SD (n = 9 per group). C = control group; LPS 1 = lipopolysaccharide 1 mg/kg + saline; D1 = lipopolysaccharide 1 mg/kg + donepezil 1 mg/kg; D3 = lipopolysaccharide 1 mg/kg + donepezil 3 mg/kg; T3 = lipopolysaccharide 1 mg/kg + tacrine 3 mg/kg; T5 = lipopolysaccharide 1 mg/kg + tacrine 5 mg/kg. Brackets indicate comparisons against the LPS 1 group, and asterisks denote statistical significance (* *p* < 0.05, *** *p* < 0.001).

Recall-phase U/K entry ratio values are summarized in Figure 11.

One-way ANOVA revealed a significant overall treatment effect on this parameter (F(5,48) = 4.43, *p* = 0.0021). Mean values were 1.021 ± 0.392 in the control group, 0.602 ± 0.252 in the LPS 1 group, 1.607 ± 0.623 in the D1 group, 1.681 ± 1.013 in the D3 group, 1.544 ± 0.814 in the T3 group, and 2.078 ± 1.037 in the T5 group. In Dunnett’s post hoc comparisons using LPS 1 as the reference condition, significantly higher U/K entry ratio values were observed in D1 (*p* = 0.0271), D3 (*p* = 0.0157), T3 (*p* = 0.0423), and T5 (*p* = 0.0006), whereas the difference between C and LPS 1 was not statistically significant (*p* = 0.6440).

These findings indicate that the treatment groups showed a more novelty-directed entry pattern during recall than the LPS 1 group, as reflected by higher U/K entry ratio values.

#### 3.2.7. Mean Time per Entry

Table 4 shows MTPE for the K and U arms during recall. One-way ANOVA did not reveal a significant overall treatment effect for MTPE into the K arm (F(5,48) = 0.29, *p* = 0.9139) or into the U arm (F(5,48) = 0.56, *p* = 0.7270). These findings suggest that the between-group differences observed in recall-phase arm times and entries were not accompanied by significant changes in average dwell time per visit.

## 4. Discussion

The present study evaluated the effects of donepezil and tacrine on recall-related exploratory behavior in a subacute lipopolysaccharide-induced model of neuroinflammation-associated cognitive impairment. The findings show that repeated LPS administration produced a consistent recall-phase behavioral alteration, reflected by reduced novelty-directed exploration and lower U/K-based indices. In the intervention experiment, cholinesterase inhibition attenuated several components of this LPS-induced behavioral alteration, with donepezil and tacrine showing partially distinct response patterns across time-based, entry-based, and ratio-derived measures.

For model validation, the primary recall-phase endpoint was the U/K time ratio, because it reflects the relative allocation of exploration between the unknown and known arms at the individual-animal level. Repeated LPS administration significantly reduced this ratio, indicating a shift of recall-phase exploration away from the unknown arm and toward the known arm. This finding suggests that the subacute LPS protocol produced a measurable reduction in novelty-directed recall behavior. This interpretation was supported by the secondary outcomes. LPS-treated rats showed reduced time and entry discrimination indices, spent less time in the unknown arm, and made fewer entries into this arm during recall. In contrast, acquisition-phase parameters, total arm entries, and mean time per entry were not significantly altered, suggesting that the observed recall deficit was not primarily driven by differences in baseline exploration, global locomotor activity, or average dwell time per visit.

These findings are consistent with previous studies showing that repeated systemic LPS administration can alter cognitive performance across different behavioral paradigms [8]. In rat models using LPS at 1 mg/kg i.p. for several consecutive days, LPS exposure impaired spatial learning and memory in the Morris water maze and was associated with hippocampal inflammatory, oxidative, apoptotic, and histopathological alterations [8,38,39]. The present findings complement this evidence by showing that a similar subacute inflammatory challenge also modifies the distribution of exploration during the 24 h Y-maze recall session. This adds a recall-focused behavioral dimension to previous LPS-based studies that primarily assessed spatial learning, working memory, or hippocampal histological changes using paradigms such as the Morris water maze and spontaneous alternation Y-maze [32].

In the intervention protocol, the U/K time ratio provided the main reference for evaluating treatment effects in relation to the LPS-induced deficit identified during model validation. Compared with the LPS 1 group, donepezil at 1 mg/kg and tacrine at 5 mg/kg significantly increased the U/K time ratio, indicating a shift toward novelty-directed exploratory allocation during recall. Although donepezil 3 mg/kg and tacrine 3 mg/kg did not reach significance for the primary ratio, their effects were further considered in relation to the supportive outcomes.

These supportive measures refined the interpretation of the primary result. Both time- and entry-based discrimination indices were significantly higher in all treated groups compared with LPS 1, indicating a more novelty-oriented recall profile after cholinesterase inhibition. Absolute arm-time analysis showed that all treated groups spent less time in the familiar arm than LPS 1, whereas the clearest increases in unknown-arm time were observed in the D3 and T5 groups. Entry-based measures showed a similar redistribution, with increased entries into the unknown arm in treated animals while total arm entries remained unchanged. In addition, MTPE values were comparable among groups, suggesting that treatment-related changes were not primarily explained by altered average dwell time per visit.

Overall, the intervention experiment suggests that cholinesterase inhibition modified the LPS-related recall phenotype mainly by changing arm-selection and revisiting patterns, rather than by producing a generalized increase in exploratory activity.

The novelty of the present approach should be interpreted in relation to previous LPS-based behavioral studies and cholinesterase inhibitor investigations in inflammatory models. Previous LPS-based studies have evaluated cognitive impairment using paradigms such as the Morris water maze and spontaneous alternation Y-maze [8,32]. Earlier studies also evaluated anti-dementia drugs, including tacrine, rivastigmine, and donepezil, in LPS-induced neuroinflammatory models, but mainly through biochemical endpoints such as IL-2 levels and acetylcholinesterase activity [43]. Other studies investigated donepezil in LPS- or Aβ-stimulated neuroinflammatory settings, focusing primarily on microglial activation, cytokine expression, and intracellular signaling pathways [25]. In contrast, the present study focused on recall-phase exploratory organization in a two-trial Y-maze paradigm and directly compared donepezil and tacrine under the same subacute systemic LPS challenge. Thus, the novelty of the present work resides not in the use of LPS or cholinesterase inhibitors separately, but in integrating a subacute inflammatory challenge with a recall-focused exploratory analysis and a direct behavioral comparison between two pharmacologically distinct cholinesterase inhibitors.

At the same time, these findings should be interpreted primarily within a behavioral pharmacology framework. Because donepezil-only and tacrine-only groups were not included, these effects should be interpreted as treatment-related differences under LPS challenge rather than as intrinsic behavioral profiles of donepezil or tacrine in non-LPS-treated animals. The study indicates that subacute systemic LPS administration modifies recall-related exploratory allocation in the Y-maze and that donepezil and tacrine can differentially influence this profile. Because the present analysis focused on behavioral endpoints, the results provide functional evidence of treatment-related modulation of the LPS-induced recall phenotype, while the underlying inflammatory, cholinergic, oxidative, and cellular mechanisms remain to be clarified in future studies.

From a pharmacological perspective, the observed effects are compatible with the established role of cholinergic signaling in memory-related processes and exploratory behavior [21,22]. Although donepezil and tacrine both enhance cholinergic neurotransmission, their distinct pharmacological profiles may contribute to different patterns of behavioral modulation under inflammatory challenge conditions [24,29,30,31]. In clinical terms, donepezil remains a currently used symptomatic cholinesterase inhibitor [24], whereas tacrine has mainly historical clinical relevance as the first approved cholinesterase inhibitor [29], while remaining pharmacologically important as a reference scaffold for tacrine-derived multitarget anti-Alzheimer compounds [30,31]. Therefore, the present comparison should be understood as an experimental pharmacology comparison rather than as a direct reproduction of clinical efficacy.

From a practical and translational perspective, the present protocol may serve as a preclinical screening framework for detecting treatment-related modulation of inflammation-associated recall deficits. By combining subacute systemic LPS exposure with a two-trial Y-maze recall task, this approach allows novelty-directed exploratory allocation to be assessed while also monitoring total activity and mean time per entry. This may help guide future studies of cholinergic and anti-inflammatory strategies in neuroinflammation-associated cognitive impairment.

The non-linear pattern observed with donepezil is also noteworthy. Although donepezil 1 mg/kg significantly improved the primary U/K time ratio, donepezil 3 mg/kg did not reach significance for this parameter, despite showing favorable effects on discrimination indices and unknown-arm time. This suggests that the relationship between cholinergic enhancement and recall-related exploratory behavior may not be strictly dose-dependent. Greater cholinergic stimulation may not necessarily translate into proportional behavioral improvement, particularly in a neuroinflammatory context where performance may depend on the balance between cholinergic modulation, arousal, exploratory drive, and inflammatory signaling.

The tacrine profile should also be interpreted with caution. Although the present findings are compatible with a role for broader cholinesterase inhibition, tacrine is pharmacologically complex and has been associated with additional actions involving cholinergic receptor systems, monoamine oxidase activity, and ion-channel function [44,45,46,47]. Therefore, the stronger effect observed at 5 mg/kg should not be attributed exclusively to acetylcholinesterase/butyrylcholinesterase inhibition.

Compared with our previous trihexyphenidyl-based Y-maze study, the present LPS-induced model showed a different response pattern to donepezil and tacrine, suggesting that the behavioral effects of cholinesterase inhibition may depend on the impairment-inducing agent [37]. This comparison supports the complementary value of inflammatory challenge and antimuscarinic models, as they address different experimentally induced aspects of Alzheimer-like pathology.

Future studies should combine the present behavioral approach with biochemical, molecular, and histological endpoints to confirm the inflammatory status induced by LPS and clarify the mechanisms through which donepezil and tacrine modify recall-related exploratory behavior. In addition, future work could explore whether LPS-induced recall alterations interact with broader neuroimmune and neuromodulatory pathways, including microbiota–gut–brain axis mechanisms, which have been increasingly discussed as modulators of neuroinflammation, neurotransmitter signaling, and cognition-related behavior [48].

## 5. Limitations

Several limitations should be acknowledged. First, the two-trial Y-maze provides useful information on recall-related exploratory allocation, but performance in this task may also reflect factors other than memory, such as anxiety-like behavior, novelty preference, arousal or residual sickness-related effects following inflammatory challenge. The lack of significant differences in acquisition-phase behavior, total arm entries, and mean time per entry argues against a major nonspecific reduction in exploration, but complementary tests such as the open-field, elevated plus maze, or additional memory paradigms would help clarify the contribution of affective and locomotor factors.

Although animals were housed individually, cages were placed adjacent to one another in the same room, allowing indirect sensory contact between animals while preventing direct physical interaction. Nevertheless, the absence of direct cage-mate contact may represent a background factor potentially influencing stress-related behavioral or neuroimmune responses. Since this condition was applied uniformly to all groups, relative between-group comparisons remain interpretable, but future studies could further examine whether housing conditions modulate the LPS-induced recall phenotype.

Second, the present study relied on behavioral endpoints and did not include biochemical, molecular, or histological validation of neuroinflammation or cholinesterase-related mechanisms. Accordingly, the findings should be interpreted as behavioral evidence obtained under an LPS-induced inflammatory challenge, while the specific inflammatory, oxidative, cholinergic, and cellular mechanisms remain to be confirmed in future studies combining behavioral testing with biochemical, molecular, and histological endpoints.

Third, the intervention protocol did not include groups receiving donepezil or tacrine in the absence of LPS. As a result, treatment-related effects should be interpreted as effects observed under LPS challenge, and cannot be fully separated from the intrinsic behavioral effects of these compounds in non-LPS-treated animals. Future studies should include drug-only groups to better distinguish baseline drug effects from effects expressed under inflammatory challenge conditions.

Finally, the study used a moderate sample size and a subacute systemic LPS protocol. Although this approach is suitable for modeling selected inflammation-associated cognitive and exploratory alterations, it cannot capture the long-term, progressive, and multifactorial nature of Alzheimer’s disease. Because ratio-based measures may also be influenced by denominator variability, interpretation should rely on the convergence of the primary U/K time ratio with supportive time-, entry-, discrimination-, and time-per-entry outcomes.

## 6. Conclusions

In conclusion, systemic LPS administration at 1 mg/kg altered exploratory behavior during the 24 h recall session of the two-trial Y-maze, mainly by reducing novelty-directed exploration. Under the same experimental conditions, donepezil and tacrine attenuated several components of the LPS-related behavioral profile, with partially distinct patterns across primary and supportive outcomes. These findings support the usefulness of this subacute LPS model for evaluating behavioral pharmacological modulation of recall-related exploratory behavior. However, further biochemical, molecular, and histological validation is required to clarify the inflammatory, oxidative, cholinergic, and cellular mechanisms underlying the observed effects.

## Figures and Tables

**Table 1:** Behavioral parameters assessed during session 1 (mean ± SD, n = 9 per group). C = control group; LPS 1 = lipopolysaccharide 1 mg/kg.

Arm	C	LPS 1
S-arm time (s)	95.00 ± 34.67	105.44 ± 34.07
K-arm time (s)	119.67 ± 29.51	107.89 ± 28.07
S-arm entries	6.67 ± 2.00	6.78 ± 1.99
K-arm entries	7.78 ± 1.56	6.33 ± 1.87

**Table 2:** MTPE in the K and U arms during the recall trial (mean ± SD, n = 9 per group). C = control group; LPS 1 = lipopolysaccharide 1 mg/kg.

Arm	C	LPS 1
U-arm (s)	17.37 ± 9.92	18.48 ± 12.96
K-arm (s)	14.74 ± 7.55	12.43 ± 5.62

**Table 3:** Behavioral parameters recorded during the acquisition session (mean ± SD, n = 9 per group). C = control group; LPS 1 = lipopolysaccharide 1 mg/kg; D1 = LPS 1 mg/kg + donepezil 1 mg/kg; D3 = LPS 1 mg/kg + donepezil 3 mg/kg; T3 = LPS 1 mg/kg + tacrine 3 mg/kg; T5 = LPS 1 mg/kg + tacrine 5 mg/kg.

Arm	C	LPS 1	D1	D3	T3	T5
S-arm time (s)	95.67 ± 33.45	98.44 ± 39.25	107.56 ± 34.66	105.00 ± 30.34	100.33 ± 33.51	96.78 ± 38.95
K-arm time (s)	113.22 ± 24.80	104.11 ± 28.70	112.67 ± 28.18	116.00 ± 19.54	119.11 ± 22.50	109.56 ± 34.98
S-arm entries	5.89 ± 1.90	6.22 ± 3.19	7.44 ± 2.88	7.22 ± 1.64	6.56 ± 2.46	6.11 ± 3.41
K-arm entries	7.00 ± 3.00	7.44 ± 1.74	6.56 ± 3.21	7.44 ± 2.88	6.56 ± 2.19	8.22 ± 2.54

**Table 4:** MTPE in the K and U arms during the recall trial (mean ± SD, n = 9 per group). C = control group; LPS 1 = lipopolysaccharide 1 mg/kg; D1 = lipopolysaccharide 1 mg/kg + donepezil 1 mg/kg; D3 = lipopolysaccharide 1 mg/kg + donepezil 3 mg/kg; T3 = lipopolysaccharide 1 mg/kg + tacrine 3 mg/kg; T5 = lipopolysaccharide 1 mg/kg + tacrine 5 mg/kg.

Arm	C	LPS 1	D1	D3	T3	T5
U-arm (s)	16.23 ± 3.78	17.79 ± 6.46	17.20 ± 12.32	15.06 ± 7.28	13.52 ± 5.18	13.76 ± 3.79
K-arm (s)	14.06 ± 5.71	15.86 ± 5.92	15.77 ± 5.93	13.98 ± 5.49	15.72 ± 5.79	17.33 ± 11.20

## Data Availability

The original contributions presented in this study are included in the article. Further inquiries can be directed to the corresponding authors.

## Abbreviations

AD	Alzheimer’s disease
LPS	lipopolysaccharide
AChE	acetylcholinesterase
BuChE	butyrylcholinesterase
i.p.	intraperitoneal
ARRIVE	Animal Research: Reporting of In Vivo Experiments
SD	standard deviation
ANOVA	analysis of variance
MTPE	mean time per entry
U arm	novel/unknown arm
K arm	familiar/known arm
S arm	start arm
